# Patterns of Virus Exposure and Presumed Household Transmission among Persons with Coronavirus Disease, United States, January–April 2020

**DOI:** 10.3201/eid2709.204577

**Published:** 2021-09

**Authors:** Rachel M. Burke, Laura Calderwood, Marie E. Killerby, Candace E. Ashworth, Abby L. Berns, Skyler Brennan, Jonathan M. Bressler, Laurel Harduar Morano, Nathaniel M. Lewis, Tiffanie M. Markus, Suzanne M. Newton, Jennifer S. Read, Tamara Rissman, Joanne Taylor, Jacqueline E. Tate, Claire M. Midgley, for the COVID-19 Case Investigation Form Working Group

**Affiliations:** Centers for Disease Control and Prevention, Atlanta, Georgia, USA (R.M. Burke, M.E. Killerby, L. Harduar Morano. N.M. Lewis, S.M. Newton, J. Taylor, J.E. Tate, C.M. Midgley);; Cherokee Nation Assurance, Arlington, Virginia, USA (L. Calderwood),; Virginia Department of Health, Richmond, Virginia, USA (C.E. Ashworth);; Rhode Island Department of Health, Providence, Rhode Island, USA (A.L. Berns);; Georgia Department of Health, Atlanta (S. Brennan);; Alaska Department of Health and Social Services, Anchorage, Alaska, USA (J.M. Bressler);; Pennsylvania Department of Health, Harrisburg, Pennsylvania, USA (L. Harduar Morano);; Vanderbilt University Medical Center, Nashville, Tennessee, USA (T.M. Markus);; Vermont Department of Health, Burlington, Vermont, USA (J. Read);; University of Vermont, Burlington (J.S. Read);; Yale School of Public Health, New Haven, Connecticut, USA (T. Rissman)

**Keywords:** 2019 novel coronavirus disease, coronavirus disease, COVID-19, severe acute respiratory syndrome coronavirus 2, SARS-CoV-2, viruses, respiratory infections, zoonoses, household transmission, United States

## Abstract

We characterized common exposures reported by a convenience sample of 202 US patients with coronavirus disease during January–April 2020 and identified factors associated with presumed household transmission. The most commonly reported settings of known exposure were households and healthcare facilities; among case-patients who had known contact with a confirmed case-patient compared with those who did not, healthcare occupations were more common. Among case-patients without known contact, use of public transportation was more common. Within the household, presumed transmission was highest from older (>65 years) index case-patients and from children to parents, independent of index case-patient age. These findings may inform guidance for limiting transmission and emphasize the value of testing to identify community-acquired infections.

Coronavirus disease (COVID-19) was first identified in Wuhan, China, in December 2019 (*1*). The first reported case in the United States was identified in January 2020 (*2*); by mid-March, cases had been reported in all 50 states (*3*). On March 16, 2020, the White House Coronavirus Task Force published guidance for curbing community spread of COVID-19 (*4*); soon after, states began to enact stay-at-home orders (*5*). By late May 2020, all 50 states had begun easing restrictions; reported cases reached new peaks in the summer and then winter months of 2020 (*6*,*7*). As restrictions further ease with increased availability of vaccine, and as pandemic fatigue may cause persons to adhere less consistently to recommended guidance such as masking and distancing, it may be informative to look back at exposures and within-household transmission during a period when few mitigation measures were in place. We characterized exposures common among persons with the earliest reported confirmed COVID-19 cases in the United States (onset mid-January through early April 2020) and identified factors associated with presumed household transmission.

This activity was reviewed by the Centers for Disease Control and Prevention (CDC) and was conducted consistent with applicable federal law and CDC policy. Forms were approved under the Office of Management and Budget (no. 0920–1011).

## Methods

### Data Source

The case investigation form (CIF) is a supplemental questionnaire designed by CDC in January 2020 to collect detailed demographic and epidemiologic information about a convenience sample of US COVID-19 case-patients reported by participating states. This purposive nonprobability sample was selected at the state level from persons identified through care-seeking, surveillance, or contact tracing as having COVID-19; infection with severe acute respiratory coronavirus 2 (SARS-CoV-2) was confirmed by reverse transcription PCR. CDC provided guidance for selection of case-patients across a range of ages and symptom severities (i.e., hospitalized and nonhospitalized), but states individually controlled sampling. The CIF was completed by state or local health department personnel or by CDC staff through case-patient or proxy interviews, along with medical record reviews (when relevant).

Case-patient demographic information included age, sex, race, ethnicity, and occupation. Workplace settings were classified according to 2012 census industry codes. Clinical information included underlying conditions, symptoms, symptom onset date, dates of medical visits, and outcome (death or survival). For hospitalized case-patients, information was requested about whether the patient had been admitted to an intensive care unit, whether oxygen was received, admission and discharge dates, diagnosis, and location. Questions about exposure included whether in the 14 days before illness onset the case-patient had known exposure to a case-patient with laboratory-confirmed COVID-19 (COVID-19 contact) and, if so, the relationship and setting of the exposure. Case-patients were also asked about their exposure risks (activities and possible exposures in the 14 days before illness onset) including travel; friends, acquaintances, co-workers, or family members with fever or respiratory symptoms; close contact with (e.g., caring for, speaking with, or touching) any ill persons; attendance at a mass gathering (e.g., religious event, concert, sports event); public transportation use; attendance or work at a school or daycare; school or daycare attendance by household members; close contact with a contact of a laboratory-confirmed case-patient; close contact with someone with fever, acute respiratory illness, or both who had traveled internationally in the previous 14 days; and time in a healthcare setting as an employee, patient, or visitor.

The CIF also collected data on the case-patient’s household members, defined as anyone who stayed overnight in the same residence as the case-patient during the 14 days before the case-patient’s illness onset until the date of interview. Case-patients were asked for household members’ age, sex, relationship to the case-patient, and whether each person had “experienced fever or respiratory symptoms (e.g., cough, sore throat, etc.) within 14 days before or after the COVID-19 patient’s illness”; if yes, date of illness onset was collected. When the CIF was designed in January 2020, the most commonly reported COVID-19 signs and symptoms were fever and respiratory symptoms, and guidance for mitigation measures within households had not been widely distributed.

### Analysis of Exposures

We compared exposures between those reporting known close contact with a COVID-19 case-patient in the 14 days before illness onset and those reporting no known contact. Categorical variables were compared by using χ^2^ or Fisher exact tests, as appropriate. Continuous variables were compared by using *t* tests for normally distributed data and Wilcoxon rank sum tests otherwise. p<0.05 was considered significant. Analyses were conducted in SAS version 9.4 (https://www.sas.com) and R (https://www.r-project.org).

### Analysis of Presumed Household Transmission

We separately assessed presumed household transmission by using information about household members provided by the interviewed COVID-19 case-patient (CIF subject). In the absence of SARS-CoV-2 testing data for all household members, we used reported signs and symptoms (i.e., fever or respiratory symptoms) as a proxy for symptomatic COVID-19 infection (i.e., household transmission). We analyzed households of >2 members (including the CIF subject) if the CIF subject had experienced >1 symptom (to enable identification of the first ill person [index case-patient] in the household), and symptom status was provided for >1 other household member. We required that the earliest symptom onset date in the household be >1 calendar day before symptom onset in subsequent case-patients (to limit effect of co-exposures outside the home) and that the earliest onset date in the household be >3 days (our median serial interval) before the interview (to allow time for symptoms to develop in exposed household members). We considered presumed household transmission to have occurred if >1 household member, in addition to the CIF subject, was reported as having fever or respiratory symptoms. The person with the earliest symptom onset date in a household was considered the index case-patient, regardless whether SARS-CoV-2 testing had been performed. Any members of a given household not identified as the index case-patient are hereafter referred to as household contacts.

We calculated the overall household attack rate for symptoms as the number of symptomatic household contacts divided by the total number of household contacts with reported symptom status, with Wilson score 95% CI, and the serial interval as the time from symptom onset in the index case-patient to first symptom onset in a household contact. We investigated age and sex of the index case-patients and their contacts, household size, and relationship of the contact to the index case-patient as possible correlates of contact symptom status by using generalized estimating equation logistic regression with households as the cluster and individual symptom status as the outcome; we used an exchangeable correlation matrix and robust SEs. We excluded household contacts missing symptom status from this analysis. We examined models for collinearity and reduced if necessary. We did not include hospitalization status of the index case-patient in models because of collinearity with index case-patient age. We dichotomized contact age (<18 or >18 years) to avoid collinearity with familial relationship and index case-patient age.

To explore the validity of using reported symptom status to estimate household symptomatic attack rates, we calculated sensitivity and specificity by using a subset of households for which complete reverse transcription PCR and serologic testing data were available (*8*). We conducted a sensitivity analysis by reclassifying data according to a range of plausible misclassification rates.

## Results

### Overview of the Analysis Population

Data were collected from 16 states (Alaska, Arizona, California, Connecticut, Georgia, Hawaii, Illinois, Minnesota, Pennsylvania, Rhode Island, Tennessee, Utah, Virginia, Vermont, Washington, and Wisconsin) with 202 laboratory-confirmed COVID-19 case-patients with symptom onset during January 14–April 4, 2020. Age of COVID-19 case-patients in the sample ranged from <1 to 95 years, almost all were symptomatic (195; 97%), and 1 in 3 was hospitalized for management of COVID-19 symptoms. Of the 202 case-patients, 34 (17%) reported having diabetes mellitus and 48 (24%) reported hypertension.

### Exposures

A total of 82 (41%) case-patients reported known contact with a laboratory-confirmed COVID-19 case-patient in the 14 days before symptom onset. The most commonly reported exposure setting was the household (44/82; 54%); within the household setting, the most frequently reported source of COVID-19 exposure was the spouse or partner of the COVID-19 case-patient (16/44; 36%). The second most reported exposure setting was healthcare (20/82; 24%); 14 of the 20 persons exposed in the healthcare setting were healthcare workers, 4 were seeking care for unrelated medical issues, and 2 were visitors.

Among persons reporting no known COVID-19 contact, 20/84 (24%) reported having close contact with an ill person. Persons with no known COVID-19 contact worked in a variety of industries, most commonly healthcare (10/90; 11%); professional/office settings (10/90; 11%); education (9/90; 10%); and accommodation, food, or other services (9/90; 10%) (Table 1). In comparison, 28% (20/72) of persons with known COVID-19 contact reported working in healthcare. Persons with no known COVID-19 contact were significantly less likely than those with known contact to report spending time in a healthcare setting (p = 0.004). However, they were somewhat more likely to report travel (38% vs. 26%) or attendance at a mass gathering (36% vs. 21%) and significantly more likely to report use of public transportation (44% vs. 16%), compared with persons reporting known COVID-19 contact (p = 0.005)

**Table 1:** Reported exposures of 179 COVID-19 case-patients with submitted case investigation forms by known contact with a laboratory-confirmed COVID-19 case-patient, United States, January–April 2020*

Exposure	No known contact, no. (%), n = 97	Known contact, no. (%), n = 82	p value†
Workplace setting‡			0.10
Accommodation, food, and other services§	9 (10.0)	2 (2.8)	
Construction	4 (4.4)	1 (1.4)	
Education¶	9 (10.0)	5 (6.9)	
Healthcare	10 (11.1)	20 (27.8)	
Manufacturing	2 (2.2)	1 (1.4)	
Professional or office setting	10 (11.1)	7 (9.7)	
Transportation and warehousing and utilities	8 (8.9)	3 (4.2)	
Wholesale or retail trade	3 (3.3)	7 (9.7)	
Other	7 (7.8)	6 (8.3)	
Insufficient information	5 (5.6)	6 (8.3)	
Not currently in the workforce	23 (25.6)	14 (19.4)	
Other exposure risks in previous 14 d			
Spent time in a healthcare setting			0.0044
Yes	24 (26.1)	39 (48.1)	
No	68 (73.9)	42 (51.9)	
Close contact with a contact of a confirmed case			0.0002
Yes	3 (3.6)	17 (25.4)	
No	81 (96.4)	50 (74.6)	
Attended a mass gathering**			0.07
Yes	29 (35.8)	16 (21.3)	
No	52 (64.2)	59 (78.7)	
Used public transportation			0.0048
Yes	23 (44.2)	8 (16.3)	
No	29 (55.8)	41 (83.7)	
Attended or worked at a school or daycare			1.00
Yes	8 (14.3)	7 (14.3)	
No	48 (85.7)	42 (85.7)	
Had a household member who attended school or daycare			0.51
Yes	15 (18.3)	9 (13.0)	
No	67 (81.7)	60 (87.0)	
Travel away from home			0.14
International, with or without domestic	18 (18.9)	8 (10.0)	
Domestic only	18 (18.9)	13 (16.3)	
None	59 (62.1)	59 (73.8)	

*A total of 23 persons did not know or did not report whether they had known contact with a person with laboratory-confirmed COVID-19 in the 14 d before their own illness onset. Denominators differ because some questions had incomplete responses. All complete responses are presented for each question. COVID-19, coronavirus disease. †χ^2^ or Fisher exact test. ‡Based on 2012 census industry codes. Mapping shown in Appendix 2 (https://wwwnc.cdc.gov/EID/article/27/9/20-4577-App2.pdf). §Not including public administration services. ¶Includes persons >18 y of age who are pursuing higher education. **Examples given in the questionnaire included religious event, wedding, party, dance, concert, banquet, festival, sports event, or other event.

Of the 202 case-patients, 23 (11.3%) reported no known contact with a confirmed case-patient, no travel within 14 days before illness onset, and none of the exposure risks assessed. These persons ranged in age from 21 to 88 years and were significantly older than those reporting >1 possible exposure (median age 52 vs. 49 years; p<0.0001). They required hospitalization more frequently than those reporting >1 possible exposure (52% [12/23] vs. 30% [54/179]; p = 0.10), and were significantly more likely to report >1 underlying medical condition (87% [20/23] vs. 58% [104/179]; p = 0.029). They were much more likely to report having diabetes mellitus (43% [10/23] vs. 14% [24/176]; p = 0.002).

### Analysis of Presumed Household Transmission

**Figure 1:**
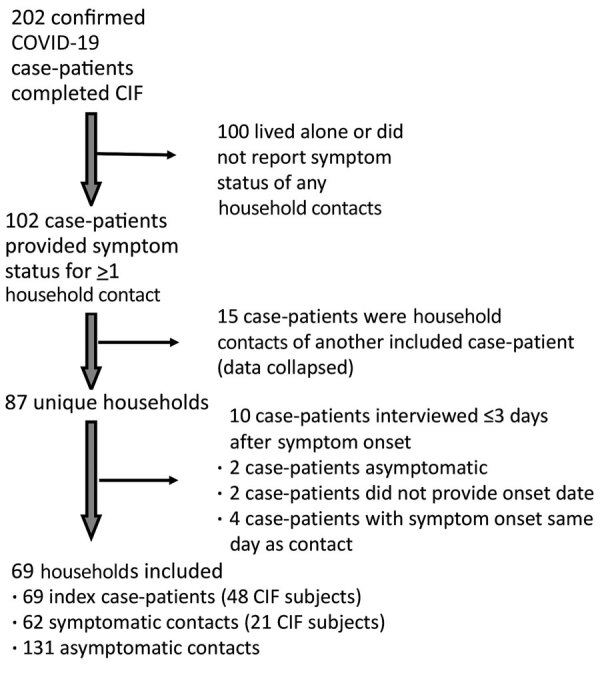
Households included in the analysis population for study of presumed household transmission among persons with COVID-19, United States, January–April 2020. CIF, case investigation form; CIF subject, interviewed COVID-19 case-patient; COVID-19, coronavirus disease.

**Figure 2:**
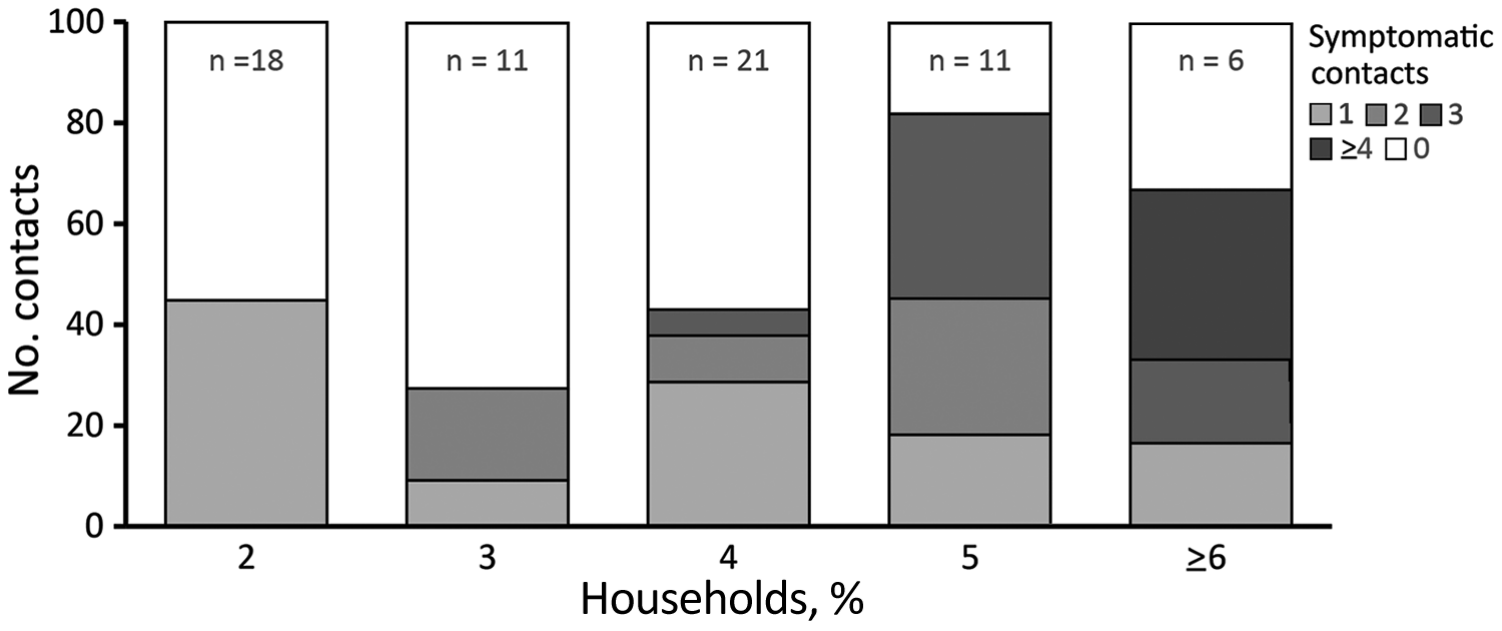
Proportion of households with presumed severe acute respiratory syndrome coronavirus 2 transmission, by household size (including index case-patient), United States, January–April 2020. Shading indicates percentage of households with the specified number of symptomatic household contacts (i.e., excluding index case-patient); households with zero symptomatic contacts (in white) are those in which presumed household transmission did not occur. n = no. households in each stratum.

A total of 69 case-patients provided data on the symptom status of >1 household members and were included in our household analysis; in 48 (70%) households, the CIF subject was the first or only symptomatic person in the household (i.e., was identified as the index case-patient; Figure 1). In half (34/69; 49%) of included households, >1 household member, in addition to the CIF subject, was symptomatic (i.e., virus transmission was presumed). Included households ranged in size from 2 to 16 persons (median 4 persons) and comprised a variety of household types (e.g., couples, nuclear families, roommates, multigenerational); household size and members’ ages, sexes, and relationships were interrelated. Presumed transmission was more frequently observed in larger households (78% of households with >5 members vs. 39% of households with <5 members; p = 0.005) (Figure 2). Within households with more members, a larger number of household contacts reported symptoms (Figure 2).

Among 201 household contacts, 193 had data on symptom status, of which 62 (32%; 95% CI 26%–39%) were symptomatic. Sensitivity analysis results showed a similar plausible range of attack rates (21%–39%). The median serial interval was 3 days (range 1–10 days).

**Figure 3:**
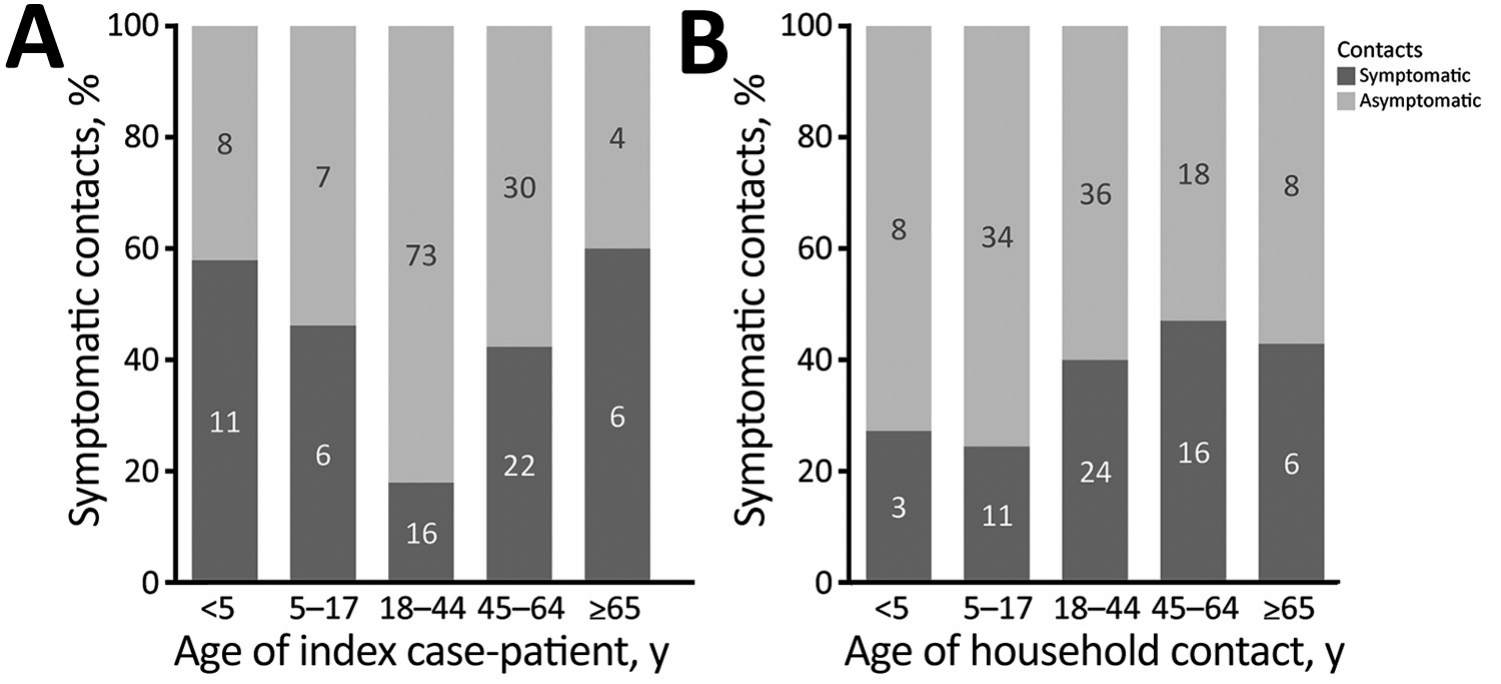
Symptom status of household contacts, by age group of index coronavirus disease case-patient (n = 192) and age group of household contact (n = 173), United States, January–April 2020. Age group missing for 20 contacts; age of index case-patient missing for 1 contact.

Although our sample did not have large numbers of index case-patients at the age extremes, household contacts were more likely to be symptomatic if the index case-patient was <5 (5 households) or >65 years of age (9 households) (Figure 3, panel A); trends were similar, but the point estimates were significant only for index case-patients >45 years of age (vs. index case-patients 18–44 years of age) after adjustment for contact age, contact sex, household size, and relationship of the contact to the index case-patient (Table 2). Adult contacts were symptomatic more often than contacts <18 years of age (Figure 3, panel B), but this association was not significant in adjusted analyses (Table 2). The symptom status of household contacts was also associated with their relationship to the index case-patient (Table 2). Among the contacts of 9 index case-patients <18 years of age, 11/16 (69%) parents, 6/13 (46%) siblings, and 2/5 (40%) other household contacts later became symptomatic. Among contacts of the 60 adult index case-patients, 12/44 (27%) children (range 2–49 years of age), 12/45 (27%) spouses/partners, 7/16 (44%) parents, and 11/42 (26%) other household contacts became symptomatic. When we restricted the analysis to households in which the CIF subject was the index case-patient, overall trends were similar to those reported above, but small sample sizes precluded adjusted analyses.

**Table 2:** Factors associated with symptom status of 172 household contacts of 64 symptomatic index case-patients in households with presumed COVID-19 transmission, United States, January–April 2020*

Factor	Unique households	No. with symptoms/no. total contacts (%)	aOR (95% CI)†	p value‡
Contact sex				0.73
F	50	28/85 (32.9)	Referent	
M	46	29/87 (33.3)	0.90 (0.49–1.64)	
Contact age, y				0.73
<18	25	13/50 (26.0)	Referent	
>18	63	44/115 (38.3)	1.15 (0.53–2.47)	
Household size, persons				0.006
<5	48	23/92 (25.0)	Referent	
>5	16	34/80 (42.5)	3.56 (1.45–8.74)	
Index case-patient age, y				0.035
<5	5	11/19 (57.9)	3.69 (0.65–20.95)	
5–17	4	6/13 (46.2)	2.09 (0.39–11.05)	
18–44	26	15/82 (18.3)	Referent	
45–64	21	20/49 (40.8)	4.61 (1.45–14.66)	
>65	8	5/9 (55.6)	15.43 (2.28–104.17)	
Relationship of contact to index case-patient				0.070
Spouse	43	11/44 (25.0)	Referent	
Child	21	11/39 (28.2)	1.78 (0.58–5.45)	
Parent	17	18/31 (58.1)	4.55 (1.22–17.00)	
Other§	23	17/58 (29.3)	1.47 (0.42–5.11)	

*A total of 21 contacts from 5 households (i.e., 5 index case-patients) are excluded because of missing data: only relationship data for 7, only sex data for 2, only index case-patient’s age for 1; only contact’s age for 5, relationship and contact age for 6. Households with presumed transmission indicates households of laboratory-confirmed COVID-19 case-patients where >1 household member exhibited symptoms; index case-patient indicates household /member with first reported onset of symptoms (regardless of laboratory confirmation); household contact indicates household member of the index case-patient. aOR, adjusted odds ratio (adjusted for all variables in the table); COVID-19, coronavirus disease. †Calculated using robust SEs. ‡Generalized Wald test. §Includes siblings, grandparents, grandchildren, friends, and any household relationship or contact other than spouse, child, or parent.

Illness severity of the index case-patient could not be assessed in multivariable models because of low sample size and correlation with age. However, among 12 household contacts of 10 index case-patients requiring hospitalization (three 18–44, five 45–64, and two index case-patients >65 years of age), only 2 were symptomatic.

## Discussion

In this convenience sample of 202 early laboratory-confirmed COVID-19 case-patients, predominantly identified before widespread mitigation measures in the United States, the most commonly reported settings of known exposure were households and healthcare facilities (primarily as a workplace). Within the household, presumed transmission by age of index case-patient followed a U-shaped pattern and was significantly higher among contacts of older (>65 years of age) index case-patients than among contacts of index case-patients 18–44 years of age. Independent of index case-patient age, parents of index case-patients were significantly more likely than other household members to report development of symptoms consistent with COVID-19.

Previous research has also found healthcare workplaces and households to be commonly reported settings of COVID-19 acquisition in the United States (*9*,*10*). In our analysis, the presumed secondary symptomatic attack rate among household members was 32%, somewhat high but consistent with estimates from previous studies, ranging from 10% to 38% (*11*–*16*; J.B. Lopez et al., unpub data, https://www.medrxiv.org/content/10.1101/2020.08.19.20177188v1). We found that presumed transmission was highest among contacts of older index case-patients (>65 years of age), even when controlling for contact age category, relationship, and household size; however, our sample size was insufficient to control for underlying conditions or hospitalization status of the index case-patient or for detailed age category of the household contact, which may have confounded this relationship because evidence suggests that older adults are more susceptible to COVID-19 (*17*). Although results were not statistically significant in adjusted analyses, we also found that contacts of index case-patients <18 years of age (especially index case-patients <5 years of age) were more likely than contacts of index case-patients 18–44 years of age to be symptomatic. Further, symptoms were significantly more likely to develop in parents of index case-patients than in other household members. This relationship was independent of index case-patient age; however, in 8 households of adult case-patients with parental household members, 6 index case-patients were <30 years of age. Higher secondary transmission to the household contacts of younger versus adult or older COVID-19 case-patients has also been reported in analyses from the United Kingdom, South Korea, and Canada (*16*; B.J. Lopez et al., unpub. data, https://www.medrxiv.org/content/10.1101/2020.08.19.20177188v1; L.A. Paul, unpub. data, https://www.medrxiv.org/content/10.1101/2021.03.29.21254565v1). These findings may be explained by the fact that SARS-CoV-2–infected children may have similar or higher viral loads than adults (*18*) and that they may have closer interaction with family members, especially parents. Parents, compared with other household members, may also play a greater role in caregiving to index case-patients, even for young adults. Conversely, in multigenerational households, adult children may act as caregivers for elderly parents, possibly exposing them before symptom onset.

A substantial proportion (60%) of case-patients in our sample did not report contact with a laboratory-confirmed COVID-19 case-patient in the 14 days before illness onset. Among case-patients without known COVID-19 contact, travel and public activities were more common, although only public transportation use was significantly higher when this group was compared with case-patients with known COVID-19 contact. Public transportation has not been identified as a major source of SARS-CoV-2 transmission (*19*–*21*), although transmission on buses, trains, and commercial flights has been reported (*19*,*22*–*26*). However, in our analysis, public transportation use might also have been more common among essential workers, those living in densely populated areas, or those with a history of travel—factors that could also increase opportunity for exposure to SARS-CoV-2 (*27*). Case-patients reporting no known source of infection, travel, or any other exposure risk factor tended to be older and to have more underlying medical conditions—particularly diabetes mellitus. Persons with concurrent conditions may be not only more susceptible to severe outcomes from COVID-19 (*28*,*29*) but also more susceptible to infection, as suggested by other analyses of SARS-CoV-2 (*8*,*30*) and Middle East respiratory syndrome coronavirus (*31*); however, more investigation is warranted.

The first limitation of our study was that the COVID-19 case-patients for whom the CIF was completed are a convenience sample of case-patients reported by 16 states during January –April 2020. Given restricted testing practices in the United States during January–March 2020, these case-patients are not representative of all US COVID-19 case-patients in terms of demographics, clinical characteristics, or exposures. Furthermore, common exposures have varied in time and geography over the course of the epidemic, and it is not possible to exclude the possibility that persons without known COVID-19 exposure had contact with an asymptomatic friend, co-worker, or family member. Our observed secondary attack rates (symptomatic persons) may also have been affected by the timing of the investigation because public awareness regarding measures to mitigate within-household transmission (e.g., isolation and mask-wearing within the home) was probably lower in the early stages of the US epidemic. Information was not collected on the specifics of known COVID-19 exposure, such as mask wearing or social distancing in the home or other exposure settings, because these were not common practices during survey design. The use of a convenience sample may have also affected findings regarding presumed household transmission, such as if selection were biased toward inclusion of more severe cases or larger investigations. 

A second limitation is that SARS-CoV-2 infection in most household members was not laboratory-confirmed, so household members with other causes of illness could have been misclassified as COVID-19 case-patients and those with asymptomatic SARS-CoV-2 infections misclassified as non–case-patients. The possibility of misclassification of children may have been higher, given that young children frequently experience respiratory symptoms (*32*) and are less likely to show symptoms of SARS-CoV-2 infection (*33*–*35*). However, overall patterns were similar when analysis was restricted to laboratory-confirmed index case-patients, and the point estimate for odds of presumed symptomatic infection among contacts of index case-patients <5 years of age versus contacts of those 18–44 years of age was similar when contacts of unconfirmed index case-patients <5 years of age were excluded. In addition, 4 of 5 households with index case-patients <5 years of age reported that >1 household member attended school or daycare in the 14 days before illness onset in the CIF subject, suggesting a possible outside source of infection. Of note, similar methods are frequently used for studies of influenza (*36*), and our observed overall symptomatic attack rate and serial interval are consistent with previous knowledge of SARS-CoV-2 transmission (*37*,*38*). It is also possible that symptoms developed in some household members after the date of interview. To limit this possibility, we excluded households in which the interview took place <3 days (median serial interval in our data) after the CIF subject’s symptom onset. Similarly, some presumed secondary case-patients may have actually been index case-patients or were co-exposed to the index case-patient; we tested exclusion of contacts with a 1-day lag in symptom onset and found similar trends, although the sample size precluded adjusted models. Previous research showing longer incubation periods for older patients suggests that households with older index patients would be less affected by such misclassification (*39*,*40*). 

Last, our sample size was limited by state capacity for participation and data completeness. We did not have sufficient sample size to control for all possible confounders, such as index case-patient signs/symptoms, clinical characteristics, or detailed contact age category, so residual confounding is possible. The lower sample size also limited the precision of our estimates.

Our findings underline the exposure risk associated with work in a healthcare setting and within the household, as previously documented (*9*,*10*). However, most case-patients in the analysis did not have known contact with a laboratory-confirmed COVID-19 case-patient, reflecting unrecognized transmission and highlighting the need for widespread testing in addition to community mitigation measures such as masking, hand hygiene, physical distancing, and limiting nonessential travel, as well as vaccination (*41*–*43*). When going out in public, persons should take preventive actions and consider the risks associated with public activities by taking into account local orders, their ability to maintain physical distance during the activity, and whether they or their household members are at risk for severe illness from COVID-19 (*41*). Everyday preventive actions also protect at-risk household members. In this analysis, presumed household transmission was common, especially from the oldest index case-patients and from children to their parents. These findings are especially relevant to the context of in-person schooling because children exposed at schools or daycare centers may introduce COVID-19 into the home. Special care must be taken to mitigate exposure risks outside the home and to protect household members at high risk for severe COVID-19, such as older persons and those with concurrent conditions. Persons with COVID-19 should follow recommendations to reduce the risk for within-household transmission, such as staying in a separate room, wearing a mask around others, practicing hand and cough hygiene, and frequently cleaning high-touch surfaces (*44*).

Appendix 1 Case investigation form used in study of patterns of virus exposure and presumed household transmission among persons with coronavirus disease, United States, January–April 2020.

Appendix 2 Supplemental methods and results for study of patterns of virus exposure and presumed household transmission among persons with coronavirus disease, United States, January–April 2020.
