# Supplementary material for: Patterns of Virus Exposure and Presumed Household Transmission among Persons with Coronavirus Disease, United States, January–April 2020
Source: Emerg Infect Dis. 2021 Sep;27(9):2323–32. doi: 10.3201/eid2709.204577 (PMC8386767; doi:10.3201/eid2709.204577)
Supplement: Appendix 1 — Case investigation form used in study of patterns of virus exposure and presumed household transmission among persons with coronavirus disease, United States, January–April 2020. [file 20-4577-Techapp-s1.pdf]

Article DOI: https://doi.org/10.3201/eid2709.204577

Patterns of Virus Exposure and Presumed Household Transmission among Persons with Coronavirus Disease, United States, January–April 2020

Appendix 1

COVID-19 Case Investigation Form

Reporting jurisdiction: _______________ Case state/local ID: _______________

Reporting health department: _______________ CDC 2019-nCoV ID: _______________

Contact ID a: _______________ NNDSS loc. rec. ID/Case ID b: _______________

a. Only complete if case-patient is a known contact of prior source case-patient. Assign Contact ID using CDC 2019-nCoV ID and sequential contact ID, e.g., Confirmed case CA102034567 has contacts CA102034567 -01 and CA102034567 -02. b. For NNDSS reporters, use GenV2 or NETSS patient identifier.

Interviewer information

Name of interviewer: Last ______________________________

First______________________________________

Affiliation/Organization: ____

Telephone ______________________________ Email

____

Date of interview: ________________(MM/DD/YYYY) Date of medical chart abstraction:

_________________ (MM/DD/YYYY)

Data sources used for this form?

☐ Case-patient interview ☐ Other interview, specify relationship to case:_______________________ ☐ Medical Chart Abstraction

Case-patient's primary language: ____________________ Was this form administered via a translator? ☐ Yes ☐ No ☐ Unknown

---

Case-patient demographic information

1. Report date to CDC (MM/DD/YYYY): ____/_____/_______
2. Under what process was the case first identified? (check all that apply): ☐ PUI/sought care for acute illness ☐ Contact tracing of case patient ☐ Surveillance system, please specify:___________________________
   ☐ EpiX notification of travelers; if checked, DGMQID_______________ ☐ Unknown

☐ Other, specify:_________________

3. Date of birth (MM/DD/YYYY): ____/_____/_______
4. Age: ____________ Age units: ☐ Years ☐ Months ☐ Days
5. Sex: ☐ Male ☐ Female ☐ Other ☐ Unknown
6. Ethnicity: ☐ Hispanic/Latino ☐ Non-Hispanic/Latino ☐ Not specified
7. Race (check all that apply): ☐ White ☐ Asian ☐ American Indian/Alaska Native ☐ Black
   ☐ Native Hawaiian/Other Pacific Islander ☐ Unknown ☐ Other, specify: _________________
8. County of Residence:________________________ State of Residence:________
9. Country of Residence: ☐ United States ☐ Other, specify_________________
10. Occupation:_______________________________________
    If student, what grade level? _______________________________________
    If child, does s/he attend day care? ☐ Yes ☐ No ☐ Unknown

Travel history

11. In the 14 days prior to illness onset, were you traveling away from your home internationally?
    ☐ Yes ☐ No ☐ Unknown

12. In the 14 days prior to illness onset, were you traveling away from your home within the United States?
☐ Yes ☐ No ☐ Unknown
13. Where did you travel 14 days prior to illness onset (list **ALL** locations, including overnight transits and layovers)?

	Departure Date (MM/DD/YYYY)	Departure city, state/province/country	Arrival Date (MM/DD/YYYY)	Arrival city, state/province/country
Trip 1				
Trip 2				
Trip 3				
Trip 4				
Trip 5				

Exposure history

14. In the **14 DAYS prior to illness**, did you have close contact with another lab-confirmed COVID-19 case-patient?

☐ Yes ☐ No ☐ Unknown Date Range: Start Date (MM/DD/YYYY) ____________

End Date (MM/DD/YYYY) ____________

15. Relationship to COVID-19 **source** case (select all that apply):
☐ Spouse/Partner ☐ Child ☐ Parent ☐ Other Family ☐ Friend ☐ HCW ☐ Co-worker
☐ Classmate ☐ Roommate ☐ Contact only – no relationship ☐ Other (specify):__________________
16. Exposure setting to the COVID-19 **source** case (select all that apply):
☐ Household ☐ Work ☐ Daycare ☐ School/University ☐ Transit ☐ Rideshare ☐ Hotel ☐ Cruise Ship
☐ Healthcare ☐ Other (specify): __________________

17. In the **14 DAYS prior to illness onset,** did you:

**Exposure**	**Answer**	**Date Range**
…have any household members, friends, acquaintances, or co-workers who had fever or respiratory symptoms (e.g. cough, sore throat etc.)?	☐ Yes ☐ No ☐ Unknown	
…have close contact (e.g. caring for, speaking with, or touching) with any ill persons?	☐ Yes ☐ No ☐ Unknown	

…attend a mass gathering (e.g., religious event, wedding, party, dance, concert, banquet, festival, sports event, or other event)?	☐ Yes ☐ No ☐ Unknown	
…use public transportation (bus, train, airplane)?	☐ Yes ☐ No ☐ Unknown	
…attend or work at a school or daycare?	☐ Yes ☐ No ☐ Unknown	
…have a household member who attended school or daycare?	☐ Yes ☐ No ☐ Unknown	
…have close contact (e.g. caring for, speaking with, or touching) with a sick person who had contact with a COVID-19 patient (i.e., secondary contact to confirmed case)?	☐ Yes ☐ No ☐ Unknown	
…have close contact (e.g. caring for, speaking with, or touching) with a person who had a fever and/or acute respiratory illness and international travel in the past 2 weeks?	☐ Yes ☐ No ☐ Unknown<br>If yes where did the person travel:____________________	

18. In the **14 DAYS prior to illness onset,** did you:

Exposure	Y/N/Unk	Facility type (Select all that apply)	Date(s) exposure occurred
Work in healthcare setting:	☐ Y ☐ N ☐ Unk<br>If yes, what was your role:<br>☐ Physician<br>☐ Nurse<br>☐ Administration staff<br>☐ Housekeeping<br>☐ Patient transport<br>☐Other, specify__________	☐ Hospital<br>☐ Urgent Care<br>☐ Doctor's office/clinic<br>☐ Dialysis unit/center<br>☐ Long Term Care Facility<br>☐ Other (specify)	
Volunteer in healthcare setting	☐ Y ☐ N ☐ Unk	☐ Hospital<br>☐ Urgent Care<br>☐ Doctor's office/clinic<br>☐ Dialysis unit/center<br>☐ Long Term Care Facility<br>☐ Other (specify)	
Have direct patient contact	☐ Y ☐ N ☐ Unk	☐ Hospital<br>☐ Urgent Care<br>☐ Doctor's office/clinic<br>☐ Dialysis unit/center<br>☐ Long Term Care Facility	

		☐ Other (specify)	
Visit healthcare setting as a patient (not just for this illness)	☐ Y ☐ N ☐ Unk	☐ Hospital ☐ Urgent Care ☐ Doctor's office/clinic ☐ Dialysis unit/center ☐ Long Term Care Facility ☐ Other (specify)	
Visit healthcare setting for any reason other than as a patient	☐ Y ☐ N ☐ Unk	☐ Hospital ☐ Urgent Care ☐ Doctor's office/clinic ☐ Dialysis unit/center ☐ Long Term Care Facility ☐ Other (specify)	
Contact with a known COVID-19 case-patient in a healthcare setting	☐ Y ☐ N ☐ Unk<br>If yes, as a<br>☐ Patient<br>☐ Visitor<br>☐ HCW	☐ Hospital ☐ Urgent Care ☐ Doctor's office/clinic ☐ Dialysis unit/center ☐ Long Term Care Facility ☐ Other (specify)	

19. Do you reside in an institutional or group setting (e.g. long-term care facility/nursing home, boarding school, college dormitory, etc.)?
    ☐ Yes ☐ No ☐ Unknown

20. How many people in total resided in your household (HH) from the 14 days prior to illness through the date of this interview (excluding you)? ________. *A household member is anyone with at least one overnight stay during the 14 days prior to patient's illness onset to the date of this interview. If patient belongs to multiple HH, group HH members by identifying the 1st HH as A, the 2nd HH as B, etc.*

HH (if case-patient belongs to >1 HH)	Relation to patient	Sex M/F	Age (specify unit as years, months, or days)	Did household member have fever or respiratory symptoms (e.g. cough, sore throat, etc.) in the ***14 days prior to patient's illness onset, during the patient's illness, or 14 days after patient's illness***?	Date of illness onset of household member (MM/DD/YYYY)
☐ A ☐ B ☐ C				☐ Y ☐ N ☐ Unk	
☐ A ☐ B ☐ C				☐ Y ☐ N ☐ Unk	
☐ A ☐ B ☐ C				☐ Y ☐ N ☐ Unk	
☐ A ☐ B ☐ C				☐ Y ☐ N ☐ Unk	
☐ A ☐ B ☐ C				☐ Y ☐ N ☐ Unk	
☐ A ☐ B ☐ C				☐ Y ☐ N ☐ Unk	
☐ A ☐ B ☐ C				☐ Y ☐ N ☐ Unk	

Symptoms

21. If symptomatic, onset date of first symptom (MM/DD/YYYY): ____/_____/_______ ☐ Unknown ☐ Asymptomatic

22. If experienced symptoms, are you ☐ Still symptomatic ☐ Unknown symptom status ☐ Symptoms resolved
    If symptoms resolved, date of symptom resolution (MM/DD/YYYY): ____/_____/_____ ☐ Unknown date

23. During this illness, did you experience any of the following symptoms?

Symptom		Symptom	
Fever ≥100.4F (38C)	☐Yes ☐No ☐Unk	Cough (new onset or worsening of chronic cough)	☐Yes ☐No ☐Unk
Highest temp________ °F		Dry	☐Yes ☐No ☐Unk
Date of onset (MM/DD/YYYY) ___/___/_______		Productive	☐Yes ☐No ☐Unk
Duration of fever ≥100.4F (38C) (days) _____________		Bloody sputum (hemoptysis)	☐Yes ☐No ☐Unk
Subjective fever (felt feverish)	☐Yes ☐No ☐Unk	Shortness of breath (dyspnea)	☐Yes ☐No ☐Unk
Chills	☐Yes ☐No ☐Unk	Wheezing	☐Yes ☐No ☐Unk
Fatigue	☐Yes ☐No ☐Unk	Chest Pain	☐Yes ☐No ☐Unk
Muscle aches (myalgia)	☐Yes ☐No ☐Unk	Abdominal pain	☐Yes ☐No ☐Unk
Rash	☐Yes ☐No ☐Unk	Vomiting	☐Yes ☐No ☐Unk
Headache	☐Yes ☐No ☐Unk	Nausea	☐Yes ☐No ☐Unk
Eye redness (conjunctivitis)	☐Yes ☐No ☐Unk	Diarrhea (≥3 loose/looser than normal stools/24hr period)	☐Yes ☐No ☐Unk
Runny nose (rhinorrhea)	☐Yes ☐No ☐Unk	Poor Feeding/Poor appetite	☐Yes ☐No ☐Unk
Sore throat	☐Yes ☐No ☐Unk	Seizures	☐Yes ☐No ☐Unk
Other, specify:	☐Yes ☐No ☐Unk	Other, specify:	☐Yes ☐No ☐Unk

Past medical history

24. Do you have any pre-existing medical conditions? ☐ Yes ☐ No ☐ Unknown

Chronic Lung Disease	☐Yes	☐No	☐Unknown	
Asthma/reactive airway disease	☐Yes	☐No	☐Unknown	
Emphysema/COPD	☐Yes	☐No	☐Unknown	
Other chronic lung disease	☐Yes	☐No	☐Unknown	(If YES, specify)
Active tuberculosis	☐Yes	☐No	☐Unknown	

Diabetes Mellitus	☐Yes	☐No	☐Unknown	
Cardiovascular disease	☐Yes	☐No	☐Unknown	
Hypertension	☐Yes	☐No	☐Unknown	
Coronary artery disease	☐Yes	☐No	☐Unknown	
Heart failure/Congestive heart failure	☐Yes	☐No	☐Unknown	
Cerebrovascular accident/Stroke	☐Yes	☐No	☐Unknown	
Congenital heart disease	☐Yes	☐No	☐Unknown	
Other	☐Yes	☐No	☐Unknown	If YES, specify: ______________________
Renal disease	☐Yes	☐No	☐Unknown	
Chronic kidney disease/insufficiency	☐Yes	☐No	☐Unknown	
End-stage renal disease	☐Yes	☐No	☐Unknown	
Dialysis	☐Yes	☐No	☐Unknown	
Other	☐Yes	☐No	☐Unknown	If YES, specify:
Liver disease	☐Yes	☐No	☐Unknown	
Alcoholic hepatitis	☐Yes	☐No	☐Unknown	
Chronic liver disease	☐Yes	☐No	☐Unknown	
Cirrhosis/End stage liver disease	☐Yes	☐No	☐Unknown	
Hepatitis B, chronic	☐Yes	☐No	☐Unknown	
Hepatitis C, chronic	☐Yes	☐No	☐Unknown	
Non-alcoholic fatty liver disease (NAFLD)/NASH	☐Yes	☐No	☐Unknown	
Other	☐Yes	☐No	☐Unknown	If YES, specify:
Immunocompromised Condition	☐Yes	☐No	☐Unknown	
HIV infection	☐Yes	☐No	☐Unknown	
AIDS or CD4 count <200	☐Yes	☐No	☐Unknown	
Solid organ transplant	☐Yes	☐No	☐Unknown	
Stem cell transplant (e.g., bone marrow transplant)	☐Yes	☐No	☐Unknown	
Cancer: current/in treatment or diagnosed in last 12 months	☐Yes	☐No	☐Unknown	
Other	☐Yes	☐No	☐Unknown	If YES, specify:
Immunosuppressive therapy	☐Yes	☐No	☐Unknown	If YES, specify:________________________ ________ ______________________________

				_______________ For what condition: _________________________ _______________________________ ______________
Neurologic/neurodevelopmental disorder	☐Yes	☐No	☐Unknown	If YES, specify:
Other chronic diseases	☐Yes	☐No	☐Unknown	If YES, specify:

25. Current height: __________ (inches) OR ___________ (cm)
26. Current weight: __________ (pounds) OR ___________ (kg)
27. If female, are you currently pregnant? ☐ Yes Weeks pregnant at illness onset__________
    ☐ No ☐ Unknown
28. If female, are you postpartum (≤6 weeks postpartum)? ☐ Yes ☐ No ☐ Unknown
29. If female, are you breastfeeding? ☐ Yes ☐ No ☐ Unknown
30. If child, is he/she being breastfed? ☐ Yes ☐ No ☐ Unknown

Social history

31. Do you currently smoke cigarettes? ☐ Yes ☐ No ☐ Unknown
    If yes, how many packs of cigarettes per day? ______ For how many years? _____
32. Have you ever smoked cigarettes? ☐ Yes ☐ No ☐ Unknown
    If yes, how many packs of cigarettes per day? ______ For how many years? _____ How long since you last smoked a cigarette? ___(m) ___(y)
33. Do you currently use e-cigarettes/vape-pen? ☐ Yes ☐ No ☐ Unknown
34. In the past year, how often did you have a drink containing alcohol?
    □ Never □ Monthly or less □ 2-4 times a month □ 2-3 times per week □ 4 or more times per week

Course of Illness

35. Do you feel back to normal? ☐ Yes ☐ No ☐ Not applicable (patient deceased) ☐ Not applicable (patient asymptomatic) ☐ Unknown
    If yes, when did you feel back to normal? _____/_____/_____ (MM/DD/YYYY)
36. Did you miss work or school for this illness? ☐ Yes ☐ No ☐ Unknown
    If yes, how many days during illness? ___________
37. Did you receive any medical care for the illness? ☐ Yes ☐ No ☐ Unknown
38. If yes, where and which dates did you seek care after this illness started (check all that apply)? [Please add extra visit dates in comments box]
    ☐ Doctor's office Date 1: ______/_____/_______ (MM/DD/YYYY) Date 2: ______/_____/_______ (MM/DD/YYYY)
    ☐ Emergency room Date 1: ______/_____/_______ (MM/DD/YYYY) Date 2: ______/_____/_______ (MM/DD/YYYY)
    ☐ Retail store/pharmacy Date 1: ______/_____/_______ (MM/DD/YYYY) Date 2: ______/_____/_______ (MM/DD/YYYY)

☐ Health department Date 1: ______/_____/_______ (MM/DD/YYYY) Date 2: ______/_____/_______ (MM/DD/YYYY)
☐ Urgent care Date 1: ______/_____/_______ (MM/DD/YYYY) Date 2: ______/_____/_______ (MM/DD/YYYY)
☐ Telephone triage line Date 1: ______/_____/_______ (MM/DD/YYYY) Date 2: ______/_____/_______ (MM/DD/YYYY)
☐ Other ___________ Date 1: ______/_____/_______ (MM/DD/YYYY) Date 2: ______/_____/_______ (MM/DD/YYYY)
☐ Unknown

39. Was the patient hospitalized? ☐ Yes ☐ No ☐ Unknown **If YES, please fill out hospitalization section below If no, skip to Question #53**
Purpose: ☐ Clinical indication ☐ No clinical indication (e.g., isolation for public health)

Hospitalization

40. Hospital name: ____ Hospital phone: __________________________
41. If yes, Admission date 1 ___/___/___ (MM/DD/YYYY) , discharge date 1 ___/___/____ (MM/DD/YYYY) ☐ Patient still hospitalized
42. To where was the patient discharged?
☐ Home ☐ Transferred to another hospital ☐ Nursing facility/rehab ☐ Hospice
☐ Other _______________ ☐ Unknown
43. If hospitalized more than once, please enter the second hospitalization's admission and discharge dates:
Hospital name: ____ Hospital phone: __________________________
Admission date 2 ______/_____/_______ (MM/DD/YYYY) Discharge date 2______/_____/_______ (MM/DD/YYYY)
☐ Patient still hospitalized
44. To where was the patient discharged?
☐ Home ☐ Transferred to another hospital ☐ Nursing facility/rehab ☐ Hospice
☐ Other _______________ ☐ Unknown
45. First recorded vital signs: Temp_________ (Unit: ☐ °F / ☐ °C) Blood pressure: _________ (systolic) /_________ (diastolic)
Heart rate: _________ Resp rate:___________
O2 Sat: ________________ (Type of support required when O2 saturation was measured:
☐ Room Air ☐ Nasal Cannula ☐ Face Mask ☐ CPAP or BIPAP ☐ High Flow Nasal Cannula ☐ Invasive mechanical ventilation
☐ Other, specify: ☐ Unknown
Fraction of Inspired Oxygen/Flow __________ ☐ % ☐ Liters/minute (LPM) ☐ Unknown ☐ NA

46. First recorded laboratory values for:

	Date (MM/DD/YYYY)	Value	Unit
White blood cell (WBC) count			☐ Cells x 109/L ☐ x 1 000/µL ☐ Other: ______
Absolute neutrophil count			☐ Cells x 109/L ☐ x 1 000/µL ☐ Other: ______
Absolute lymphocyte count			☐ Cells x 109/L ☐ x 1 000/µL ☐ Other: ______
Platelets (Plt)			☐ Cells x 109/L ☐ x 1 000/µL ☐ Other: ______
Aspartate transaminase (AST)			☐ U/L ☐ IU/L ☐ Other: ______
Alanine aminotransferase (ALT)			☐ U/L ☐ IU/L ☐ Other: ______
Lactate dehydrogenase (LDH)			☐ U/L ☐ IU/L ☐ Other: ______

47. Was the patient admitted to an intensive care unit (ICU)? ☐ Yes ☐ No ☐ Unknown

ICU admission date 1 ______/_____/_______ (MM/DD/YYYY) ICU discharge date 1 ______/_____/_______ (MM/DD/YYYY)

ICU admission date 2 ______/_____/_______ (MM/DD/YYYY) ICU discharge date 2 ______/_____/_______ (MM/DD/YYYY)

48. During hospitalization, did the patient receive...

		Start Date (MM/DD/YYYY)	Last Date (MM/DD/YYYY)	Total Days
Supplemental Oxygen?	☐ Y ☐ N ☐ Unk			
BiPap or CPAP use?	☐ Y ☐ N ☐ Unk			
High flow nasal cannula?	☐ Y ☐ N ☐ Unk			
Invasive mechanical ventilation?	☐ Y ☐ N ☐ Unk			
ECMO?	☐ Y ☐ N ☐ Unk			

49. Did the patient receive a discharge diagnosis of pneumonia (refer to clinical discharge summary)?

☐ Yes ☐ No ☐ Unknown

50. Did the patient receive a discharge diagnosis of acute respiratory distress syndrome (ARDS) (refer to clinical discharge summary)?
☐ Yes ☐ No ☐ Unknown

51. Clinical Discharge Diagnoses and ICD10 Discharge Codes

**Clinical Discharge Diagnoses**	**ICD-10-CM Code**
1.	
2.	
3.	
4.	
5.	
6.	
7.	
8.	
9.	
10.	

52. Did the patient receive any antiviral medications during hospitalization for this illness:

Medication		Dose	Frequency	Start Date (MM/DD/ YYYY)	Last Date (MM/DD/ YYYY)	Total Days
Remdesivir	☐ PO<br>☐ IV<br>☐ IM					
Other:_______________<br>_______	☐ PO<br>☐ IV<br>☐ IM					
Other:_______________<br>_______	☐ PO<br>☐ IV<br>☐ IM					

Imaging

53. Was a chest x-ray taken? ☐ Yes ☐ No ☐ Unknown

54. Were any of these chest x-rays abnormal? ☐ Yes ☐ No ☐ Unknown
Date of first abnormal chest x-ray: ______/_____/_______ (MM/DD/YYYY

55. For first abnormal chest x-ray, please check all that apply: Report not available: ☐

Air space density	☐	Cannot rule out pneumonia	☐	ARDS (acute respiratory distress syndrome)	☐	Other	☐
Air space opacity	☐	Consolidation	☐	Lung infiltrate	☐	Pleural Effusion	☐
Bronchopneumonia/pneumonia	☐	Cavitation	☐	Interstitial infiltrate	☐	Empyema	☐

56. Was a chest CT/MRI taken? ☐ Yes ☐ No ☐ Unknown

57. Were any of these chest CT/MRIs abnormal? ☐ Yes ☐ No ☐ Unknown
Date of first abnormal CT/MRI: ______/_____/_______ (MM/DD/YYYY)

58. For first abnormal chest CT/MRI, please check all that apply: Report not available: ☐

Air space density	☐	ARDS (acute respiratory distress syndrome)	☐	Empyema	☐	Englarge epiglottis	☐
Air space opacity/opacification	☐	Lung infiltrate	☐	Pneumothorax	☐	Tracheal narrowing	☐
Bronchopneumonia/pneumonia	☐	Interstitial infiltrate	☐	Pneumomediastinum	☐	Ground glass opacities	☐
Consolidation	☐	Lobar infiltrate	☐	Widened mediastinum	☐	Other	☐
Cavitation	☐	Pleural effusion	☐				

Lab Results

59. SARS-CoV-2 Testing (Please report further test results in comments)

Date of sample collection (MM/DD/YYYY)	Sample Type	Result
	☐ NP ☐ OP ☐ Sputum<br>☐ Other, specify: ____________________	☐ Pos ☐ Neg<br>☐ Inconclusive
	☐ NP ☐ OP ☐ Sputum<br>☐ Other, specify: ____________________	☐ Pos ☐ Neg<br>☐ Inconclusive
	☐ NP ☐ OP ☐ Sputum<br>☐ Other, specify: ____________________	☐ Pos ☐ Neg<br>☐ Inconclusive
	☐ NP ☐ OP ☐ Sputum<br>☐ Other, specify: ____________________	☐ Pos ☐ Neg<br>☐ Inconclusive
	☐ NP ☐ OP ☐ Sputum<br>☐ Other, specify: ____________________	☐ Pos ☐ Neg<br>☐ Inconclusive

60. Was patient tested for other viral respiratory pathogens during their illness? ☐ Yes (report results below) ☐ No ☐ Unknown

	Positive	Negative	Not Tested/ Unknown	Collection Date (MM/DD/YYY)	Specimen Type
Flu A/H1	☐	☐	☐	____/____/_________	
Flu A/H3	☐	☐	☐	____/____/_________	
Flu B	☐	☐	☐	____/____/_________	
Flu (no type)	☐	☐	☐		

Respiratory syncytial virus/RSV	☐	☐	☐	____/____/________	
Adenovirus	☐	☐	☐	____/____/________	
Parainfluenza virus 1	☐	☐	☐	____/____/________	
Parainfluenza virus 2	☐	☐	☐	____/____/________	
Parainfluenza virus 3	☐	☐	☐	____/____/________	
Parainfluenza virus 4	☐	☐	☐	____/____/________	
Respiratory syncytial virus/RSV	☐	☐	☐	____/____/________	
Human metapneumovirus	☐	☐	☐	____/____/________	
Rhinovirus/enterovirus	☐	☐	☐	____/____/________	
Human coronavirus 229E	☐	☐	☐	____/____/________	
Human coronavirus HKU1	☐	☐	☐	____/____/________	
Human coronavirus NL63	☐	☐	☐	____/____/________	
Human coronavirus OC43	☐	☐	☐	____/____/________	

61. Were any bacterial culture tests performed during their illness? ☐ Yes ☐ No ☐ Unknown

If yes, was there a positive culture for a bacterial pathogen? ☐ Yes ☐ No ☐ Unknown

If yes, specify pathogen: ____

If yes, specify date of culture (MM/DD/YYYY): ____________

If yes, site where pathogen identified: ☐Blood ☐Sputum ☐Bronchoalveolar lavage (BAL) ☐Endotracheal aspirate ☐Pleural fluid ☐ Cerebrospinal fluid (CSF) ☐Other, specify: ______________

If more than one bacterial culture test was performed, please record in additional comments.

Outcome

62. Did the patient die as a result of this illness?

☐ Yes, Date: _____/_____/_____ (MM/DD/YYYY) ☐ No ☐ Unknown

Where did the death occur: ☐ Home ☐ Hospital ☐ ER ☐ Hospice ☐ Other, specify______________________________

(If the following information is not currently available, please send an update later using death certificate or death note in hospital record.)

Contribution of COVID-19 to death ☐ Underlying/primary ☐ Contributing/secondary ☐ No contribution to death ☐ Unknown

Was autopsy performed? ☐ Yes ☐ No ☐ Unknown

Primary Cause of death (death certificate/coroner)

____

Any additional comments or notes?

This is the end of the case investigation form. Thank you very much for your time. If you have any questions please feel free to contact the CDC at 770-488-7100 or eocreport@cdc.gov
