# Supplementary material for: Patterns of Virus Exposure and Presumed Household Transmission among Persons with Coronavirus Disease, United States, January–April 2020
Source: Emerg Infect Dis. 2021 Sep;27(9):2323–32. doi: 10.3201/eid2709.204577 (PMC8386767; doi:10.3201/eid2709.204577)
Supplement: Appendix 2 — Supplemental methods and results for study of patterns of virus exposure and presumed household transmission among persons with coronavirus disease, United States, January–April 2020. [file 20-4577-Techapp-s2.pdf]

Article DOI: https://doi.org/10.3201/eid2709.204577

Patterns of Virus Exposure and Presumed Household Transmission among Persons with Coronavirus Disease, United States, January–April 2020

Appendix 2

Supplemental Methods

Workplace Setting Classification

The CIF asked participants to classify their "occupation." This free text was then processed by the National Institute for Occupational Safety and Health (NIOSH) using the NIOSH Industry and Occupation Computerized Coding System (NIOCCS) to produce 2012 Census Industry Codes. Workplace settings were categorized according to 2012 Census Industry Codes, because the CIF did not ask about occupation and industry separately. The following groups were created: accommodation, food, and other services (census industry codes 8660 – 8690 or 8770 – 9290; does not include public administration); construction (census industry code 0770); education (free text of "student" among persons ≥18 years [and census industry code 9890], or census industry codes 7860 – 7890); healthcare (reported occupation as a healthcare worker or census industry codes 7970 – 8270); manufacturing (census industry codes 1070 – 3990); professional or office setting (census industry codes 6470 – 6780 or 6870 – 7190 or 7270 – 7490); transportation, warehousing, and utilities (census industry codes 0570 – 0690 or 6070 – 6390); wholesale or retail trade (census industry codes 4070 – 4590 or 4670 – 5790); insufficient information (census industry code 9990 or unable to classify industry); not currently in workforce (retired, homemaker, unemployed, child <18 years of age); other (census industry codes not previously mentioned).

Sensitivity Analysis

A subset of 18 households included in our analysis participated in a household transmission study in Utah (*1*). Laboratory-confirmed COVID-19 case-patients were identified

by public health surveillance, and their households were enrolled within 10 days of sample collection from that initial case-patient. Nasopharyngeal (NP) and serum samples were collected from all household members at enrollment and after a 14-day follow-up period and were tested for SARS-CoV-2 by RT-PCR (NP samples) and enzyme immunoassay (serum samples). Reported household member symptom status was compared to test results (counting any RT-PCR or serology positive as a confirmed COVID-19 case patient) to calculate the sensitivity and specificity of the CIF question regarding household contact symptom status ("Did household member have fever or respiratory symptoms (e.g. cough, sore throat, etc.) in the 14 days prior to patient's illness onset, during the patient's illness, or 14 days after patient's illness?"). Misclassification-adjusted attack rates were calculated for a range of the estimated sensitivity (Se) and specificity (Sp), plus or minus 10%, in increments of 5%, using the formula (*2*):

$$Adjusted\ Attack\ Rate = \frac{Symptomatic\ contacts\ -\ Total\ contacts * (1 - Sp)}{Se - (1 - Sp)} \div Total\ contacts$$

Supplemental Results

In the subset of households for whom testing data was available on all household members (*3*), 13 of 18 test-positives were identified as symptomatic (sensitivity = 72%) and 50 of 59 test-negatives were identified as asymptomatic (specificity = 85%). The misclassification-adjusted household attack rate was 30.0% (unadjusted AR = 32.1%). The adjusted attack rates for a range of sensitivity and specificity values are shown in Appendix Table 1. The most plausible values are considered to be those estimated for Sp and Se within 5% of the calculated values and are highlighted in grey. Sample-size limitations precluded age-specific sensitivity analyses.

Reference

1. Lewis NM, Chu VT, Ye D, Conners EE, Gharpure R, Laws RL, et al. Household transmission of SARS-CoV-2 in the United States. Clin Infect Dis. 2020;ciaa1166. PubMed https://doi.org/10.1093/cid/ciaa1166

2. Lash TLFM, Fink AK. Applying quantitative bias analysis to epidemiologic data: Springer; 2009.

3. Centers for Disease Control and Prevention. COVID data tracker [cited 2021 Apr 21]. https://covid.cdc.gov/covid-data-tracker/#datatracker-home.

Appendix Table 1. Misclassification-adjusted household attack rates for varying levels of sensitivity and specificity of household case identification

	Sensitivity				
Specificity	62%	67%	72%	77%	82%
75%	19.3%	17.0%	15.2%	13.7%	12.5%
80%	28.9%	25.8%	23.3%	21.3%	19.6%
85%	36.4%	32.9%	30.0%	27.6%	25.6%
90%	42.5%	38.8%	35.7%	33.0%	30.7%
95%	47.6%	43.7%	40.5%	37.7%	35.2%

Appendix Table 2. Factors associated with symptom status of 112 household contacts of 44 laboratory-confirmed index COVID-19 case patients–United States, January – April 2020*

Factor	Unique households	N with symptoms / Total contacts (%)	Crude OR	95% CI†	p-value‡
Contact Sex					
Female	37	11 / 57 (19.3%)	1.00	-	-
Male	29	7 / 55 (12.7%)	0.57	(0.24, 1.35)	0.20
Contact Age					
<18 years	17	6 / 37 (16.2%)	1.00	-	-
18+ years	43	12 / 69 (17.4%)	0.92	(0.31, 2.79)	0.89
Household Size					
<5 people	36	9 / 70 (12.9%)	1.00	-	-
5+ people	8	9 / 42 (21.4%)	2.44	(0.63, 9.47)	0.20
Index Age					
<5 years	2	2 / 7 (28.6%)			
5 - 17 years	2	0 / 5 (0.0%)			
18 - 44 years	20	8 / 65 (12.3%)		Could not calculate	
45 - 64 years	15	7 / 30 (23.3%)			
65+ years	5	1 / 5 (20.0%)			
Relationship of Contact to Index Case					
Spouse	34	4 / 35 (11.4%)	1.00	-	
Child	16	7 / 30 (23.3%)	2.68	(0.74, 9.72)	0.20
Parent	9	5 / 17 (29.4%)	2.83	(0.51, 15.76)	
Other	13	2 / 30 (6.7%)	0.73	(0.13, 4.05)	

*60 contacts from 20 households (i.e., 20 index cases) with complete data are excluded because the index case was not the subject of the CIF (i.e., was not necessarily laboratory-confirmed as SARS-CoV-2–positive). An additional 4 contacts from 1 household (i.e., 1 index case) are excluded because the index case was not the subject of the CIF and data were missing. An additional 17 contacts from 4 households (i.e., 4 index cases) are excluded due to missing data; 2 persons missing sex, 10 missing contact age category, 11 missing relationship. Definitions: Index case – household member with first reported onset of symptoms. Household contact – household member of the index case.
Abbreviations: OR –odds ratio. CI – confidence interval. CIF – Case Investigation Form.
†Calculated using robust standard errors.
‡Generalized Wald test.

Appendix Table 3. Characteristics of 202 COVID-19 case-patients with submitted case investigation forms, United States, January 14 – April 4, 2020,

Characteristic	N (%)
Reporting Month	
January – February, 2020	23 (11.4)
March, 2020	106 (52.5)
April, 2020	73 (36.1)
Demographics	
Sex	
Female	90 (44.6)
Male	106 (52.5)
Unknown	6 (3.0)
Age (years)	
0–4	5 (2.5)
5–17	10 (5.0)
18–44	71 (35.1)
45–64	66 (32.7)
65–74	26 (12.9)
75–84	12 (5.9)
85+	5 (2.5)
Unknown	7 (3.5)
Race	
American Indian / Alaska Native	1 (0.5)
Asian	37 (18.3)
Black	12 (5.9)
Multiracial	2 (1.0)
Native Hawaiian / Other Pacific Islander	4 (2.0)
White	97 (48.0)
Other*	4 (2.0)
Unknown	45 (22.3)
Ethnicity	
Hispanic / Latino	23 (11.4)
Not Hispanic / Latino	130 (64.4)
Unknown	49 (24.3)
Behavioral History	
Smoking history	
Current	4 (2.0)
Former	31 (15.3)
Never	121 (59.9)
Unknown	46 (22.8)
Alcohol consumption	
Never	62 (30.7)
Monthly or less	25 (12.4)
At least 2x per month	38 (18.8)
Unknown	77 (38.1)
Underlying conditions	
Diabetes mellitus	
No	147 (72.8)
Yes	34 (16.8)
Unknown	21 (10.4)
Obesity (BMI ≥30)	
No	60 (29.7)
Yes	35 (17.3)
Unknown	107 (53.0)
Hypertension	
No	130 (64.4)
Yes	48 (23.8)
Unknown	24 (11.9)
Chronic respiratory condition	
No	152 (75.2)
Yes	30 (14.9)
Unknown	20 (9.9)
Renal disease	
No	167 (82.7)
Yes	14 (6.9)
Unknown	21 (10.4)
Immunosuppressive condition	
No	172 (85.1)
Yes	8 (4.0)
Unknown	22 (10.9)

Characteristic	N (%)
Clinical summary	
Symptom status	
No	6 (3.0)
Yes	195 (96.5)
Unknown	1 (0.5)
Outcome	
Deceased	6 (3.0)
Survived	158 (78.2)
Unknown	38 (18.8)
Hospitalization status	
Not hospitalized	115 (56.9)
Hospitalized for clinical management of COVID-19 symptoms	66 (32.7)
Hospitalized, unknown or other purpose (e.g., isolation)	13 (6.4)
Hospitalization unknown	8 (4.0)
Information about hospitalization†	
Discharge	
Deceased	5 (7.6)
Home	23 (34.8)
Other	2 (3.0)
Unknown	36 (54.5)
Admitted to the Intensive Care Unit	
No	26 (39.4)
Yes	34 (51.5)
Unknown	6 (9.1)
Mechanical ventilation	
No	43 (65.2)
Yes	15 (22.7)
Unknown	8 (12.1)

*All persons who indicated that none of the following racial categories applied to them: American Indian / Alaska Native, Asian, Black, Multiracial, Native Hawaiian / Other Pacific Islander, White.

†For case-patients hospitalized for clinical management of COVID-19 symptoms, N = 66.
